# Delays in Referral of Soft Tissue Sarcomas

**DOI:** 10.1155/2008/378574

**Published:** 2008-02-14

**Authors:** G. D. Johnson, G. Smith, A. Dramis, R. J. Grimer

**Affiliations:** ^1^Department of Medicine, Medical School, University of Birmingham, 33 Holloway, Northfield, Birmingham B31 1TP, UK; ^2^Department of Orthopaedic Oncology, Royal Orthopaedic Hospital, Birmingham, Bristol Road South, Northfield, Birmingham B31 2AP, UK

## Abstract

*Introduction and aims*. It is well established that soft tissue sarcomas (STSs) are more effectively treated in a specialist centre. However, delays in time taken for a patient to be referred to a specialist centre may lead to a poorer prognosis. This study aims to identify the length of these delays and where they occur. *Patients and methods*. Patients with a proven STS were included. They were recruited from both outpatient clinics and from the surgical ward of the Royal Orthopaedic Hospital (Birmingham, UK). A structured interview was used to take a detailed history of the patients' treatment pathway, before arriving at the specialist centre. Dates given were validated using the case notes. *Results*. The median time for the patient to present to a specialist centre from the onset of symptoms was 40.4 weeks. The median delay until presentation to a medical professional (patient delay) was 1.3 weeks. Median delay in referral to a specialist centre (service delay) was 25.0 weeks. *Discussion*. Medical professionals rather than patients contribute the greatest source of delay in patients reaching a specialist centre for treatment of STS. Adherence to previously published guidelines could decrease this delay for diagnosis of possible sarcoma. Steps should be taken to refer patients directly to a diagnostic centre if they have symptoms or signs suggestive of STS.

## 1. INTRODUCTION

Soft tissue sarcomas (STSs) are a rare group of cancers with approximately 2200 diagnosed in the UK every year [1]. They account for approximately 1% of all cancers and 2% of all cancer deaths [2]. Survival rates are approximately 50% at 5 years and are related to grade, depth, and size of tumour at diagnosis.

Due to the rarity and high mortality rate of STS, guidelines have been introduced by the Department of Health for their early diagnosis. The guidelines state that *any lump* that is either: ≥5 cm in size and/or deep to the fascia and/or painful and/or increasing in size should be referred to a diagnostic centre for investigation and to a surgical centre for management [3]. However, recent evidence suggests that these guidelines are not well implemented, resulting in inadequate management of these tumours and delays in referral [4–7].

In the Trent Cancer Registry region, median size at presentation to the sarcoma service was 8 cm (range: 0.3–45 cm) [8] and data from the Scandinavian Sarcoma Group Register shows a median size of 7 cm at presentation, varying with age, with a larger median tumour size in older patients [9]. At our centre, however, the median size of tumour seen at first presentation is 9 cm. Thus, patients presenting to our centre have tumours nearly twice the size recommended by the guidelines. This delay in presentation to specialist services could be due to either unwillingness of patients to present initially or to delays after inappropriate primary or secondary referrals.

Due to their rarity, referral patterns for STS are often circuitous and are manifested by delays in presentation and referral. Lack of experience with these tumours is the oft-cited reason for delayed referral practice and inadequate management. With an average General Practitioner (GP) seeing only 1 STS in 20 years of practice, this claim is easily substantiated [10]. Referral to a General or Othopaedic Surgeon often increases delays to definitive treatment as, because of their rarity, a malignant diagnosis is a low clinical suspicion. As a result, inappropriate surgical techniques are often employed: lesions may be excised under the assumption that they are benign, with inadequate surgical margins [11, 12]. A recent study found that 50% of patients referred to their Sarcoma Service had received some surgical intervention at a nonspecialist centre [11].

Several papers have described delays in referral of patients with STS for treatment. These found that medical professionals were the source of the greatest delay. However, GPs and hospital consultants were not considered separately and all the studies identified a time period which the authors thought constituted “delay,” only going on to analyse those patients with delay [4–6]. However, this fails to give an accurate impression of the actual timescale.

Current guidelines in the UK state that any patient with suspected cancer should be seen by a specialist within 2 weeks of referral [13]. At present, there is no data on delay in the UK attributable to patients, and medical professional delay is difficult to quantify, as the only UK paper on the subject judged that delay was anything longer than 3 months [6]. This study aims to bring clarity to the situation, which is essential to the development of further training of medical professionals in the UK. Recent literature suggests that obesity can cause a worse prognosis and delays in presentation in cancer patients [14]; therefore we also plan to identify whether any patient factors (BMI, Social Class and Literacy Levels) have any effect on delay in presentation or size of tumour at diagnosis.

## 2. PATIENTS AND METHOD

A total of 162 patients with a confirmed diagnosis of Soft Tissue Sarcoma were recruited from a combination of the outpatient clinic and from the surgical ward at the Royal Orthopaedic Hospital (Birmingham, UK) between January 2005 and May 2005. In order to maximise patient numbers in the study, a combination of followup patients and new referrals were included. Appropriate ethical approval was gained. Patients were consented by their doctors and the study was further explained to them by a researcher at which point they were given an explanatory letter. A structured interview was used to gather the study information, which was completed by one member of the research team.

### 2.1. Demographics

We collected standard demographic information about patient age, sex, histological diagnosis, size of tumour at presentation and which features suggestive of sarcoma from the guidelines the patient had, if any.

### 2.2. Time delay

We measured duration of symptoms before a patient presented to a medical professional (MP), by asking the patients to recall when they first noticed the lump. We asked the patients to recall the dates when they first saw a medical professional (normally a GP) and when they were referred to someone, either a district general hospital (DGH) or the Royal Orthopaedic Hospital (ROH). For those patients who were referred to a DGH, we asked them to then recall when they were then referred to the ROH.

All dates were confirmed with dates in the patients’ notes. Referral date to the ROH was the only date that could be confirmed in all cases. This was therefore used to calculate the accuracy of the dates given by each patient, by comparing the ROH referral date they specified with the actual date recorded in the notes.

Patients were asked to recall the management of the first medical professional they saw and the subsequent clinicians. As a result, we calculated the number of inappropriate and inadequate procedures performed. We also recorded whether the patient had any cross-sectional imaging or a biopsy when managed at a DGH.

### 2.3. Associations

Age, sex, patients’ postal code, and the age at which they left full-time education were recorded. Using the Townsend deprivation scale, we calculated social deprivation from a patient’s postal code; and literacy levels were calculated from the age they left full-time education.

We also recorded the patients’ height in centimetres and weight in kilos. Height was recorded at interview, as this will not have significantly changed since they first had their Soft Tissue Sarcoma. Weight was recorded from the operation record to ensure that we recorded the weight of the patient as close to diagnosis as possible. Using this information we then calculated each patient’s body mass index.

## 3. STATISTICAL ANALYSIS

Simple descriptive statistics were performed on the data. Linear regressions and correlations were performed to test for associations. It was predicted that the data would not follow a normal distribution and therefore nonparametric tests were used for comparisons (Mann-Whitney-U test). The median has been chiefly used as the method of comparison because a number of patients had excessively long delays, which have skewed the mean. However, the mean has been provided for completeness.

## 4. RESULTS

**Figure 1:**
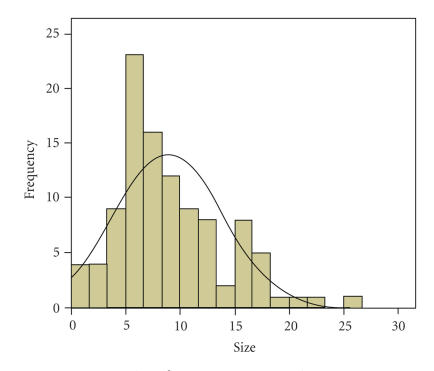
Size of tumour at presentation to ROH.

Of the 162 patients included, the mean age at diagnosis was 53 years (range 16–88). The sex distribution was 49.3% male: 50.7% female. The median size of tumour at diagnosis was 8 cm (mean 8.91 cm, range 0–26 cm) (see Figure 1). Histological subtypes followed the expected distribution with the commonest being Liposarcoma 24.1%, Myxofibrosarcoma 13.0%, and Leiomyosarcoma 7.4%. A percentage of 55.5% of lumps were deep, 43.2% high-grade, and 39.2% intermediate-grade. 88% of patients had at least one of the features that are suspicious of sarcoma at first presentation to a medical professional. Site of tumour also followed the expected distribution, with 42.5% affecting the thigh, 20% the lower leg, 14.6% the forearm, 13% the upper arm, 9.2% the trunk, and 1.5% affecting the hand.

The median time for a patient to be referred to the ROH from the onset of symptoms was 40.4 weeks (mean 112.3 weeks). Only 14.6% were referred within 3 months of the onset of symptoms and 44.9% of patients took longer than 1 year to be referred to the ROH from onset of symptoms (Table 1).

The median time for a patient to initially present to a medical professional from onset of symptoms was 1.3 weeks (mean 28.6 weeks). 60.6% of patients consulted a medical professional (91.6% of which were GPs) within 1 month. 72.5% had consulted within 3 months. 12.5% of patients waited longer than one year before consulting; the longest a patient waited before seeking medical advice was 674 weeks (13 years). From first presentation to a medical professional, the median time for a patient to be referred to the ROH was 25.0 weeks (mean 83.1 weeks). Only 11.3% of patients were referred to the ROH within 1 month and 28.9% within 3 months. 32.7% of patients took longer than 1 year to be referred to the ROH for investigation and treatment (Table 3).

 47% of patients were referred to a consultant the first time the patient presented. 51.0% were referred within 1 month and 60.9% within 3 months. 21.2% of patients took longer than 1 year to be referred. 9 of the 162 patients were referred directly to the ROH. The remaining 153 were referred to a local DGH and 37.3% of patients were referred to the ROH by the consultant they saw within 1 month. 63.3% had been referred within 3 months. 12.7% of patients managed by a consultant in a DGH took longer than 1 year to be referred to the ROH.

The 62 patients who were reassured by the first medical professional consulted took a mean of twice as long to be referred to another, more specialised, medical professional (P<.0001). The median time for a patient who had been reassured to be referred increased nearly 16 folds, to 38.3 weeks. Although the number of patients is too small for a statistically significant difference to be calculated (n=11), it appears that the decision to obtain an X-ray was related to an increased time for referral from the GP to hospital (median 6.7 weeks) (Table 2).

Performing an MRI or biopsy was related to a substantial decrease in *mean* time to refer to the ROH, although *median* time was slightly increased by performing biopsy. Performing surgery, whether or not they had an MRI &/or biopsy, showed a highly significant (P<.0001) increase in the time taken for a patient to be referred to the ROH. The 35 patients who had surgery without biopsy took nearly 3 times longer to be referred to the ROH than the study mean and median (P<.0001). The 4 patients who had surgery having already had a biopsy took a median 15.2 weeks (mean 24.3 weeks) longer, longer to be referred to the specialist centre. Only 3 patients were reassured by the DGH consultants so the difference is not statistically significant, but the mean delay was nearly 7 fold longer and median delay was nearly 27 fold longer (Table 2).

The mean BMI of the patients studied was 26.6 (range: 18–45.8). The mean age that patients had left full-time education was 16.9 and the mean Townsend score was −0.8373. No association between any of these factors and either patient delay or medical professional delay was identified. No association with BMI and size of tumour at presentation was identified. The only factor that had any correlation with delay was the size of the tumour at first presentation, where with increasing size of tumour, the time taken to refer to the ROH decreased (P=.022). There was no significant difference in time to presentation based on tumour site.

## 5. LIMITATIONS

The main limitation with this study is that it relies largely on patient recall of significant dates in their history and this is liable to bias. However, in order to minimise this effect, dates of referral given by the patient were compared in all cases with those in the notes. We found that there was no significant difference between these dates.

The population studied is an already biased population, as we only studied patients diagnosed with sarcoma in a tertiary referral centre. We are unable to take into account those who have had their sarcomas managed elsewhere.

## 6. DISCUSSION

Current guidelines state that any patient with suspected cancer should be seen by a specialist within 2 weeks of first presentation to a medical professional, normally a General Practitioner. In the case of Soft Tissue Sarcoma, patients should be referred to a specialist centre for further investigation and management.

The most recent research on delays in the referral and diagnosis of STS has identified significant delays due to medical professionals. However, these papers set a fixed time period considered as “delay” being 1 month and 3 months [4–6]. The analysis then considers only those who have been found to have a delay, thereby giving an inaccurate impression of the actually timescale in the study patients. For this analysis, no limit has been used to define “delay” and all medians and means used contain all patients, including those who did not suffer any delay.

Nearly 73% of patients presented to a medical professional (91.6% GP) within 3 months of the onset of symptoms, with a median time of 1.3 weeks (Table 3). This shows that, although some patients are more willing to tolerate symptoms, in general, patients present quickly to a medical professional and therefore do not contribute significantly to delay in reaching a specialist centre for treatment.

**Figure 2:**
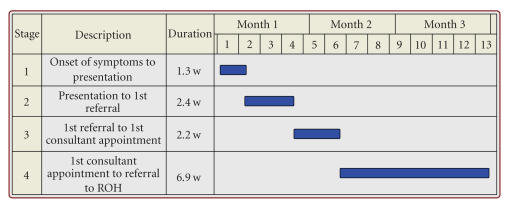
The median patient.

**Figure 3:**
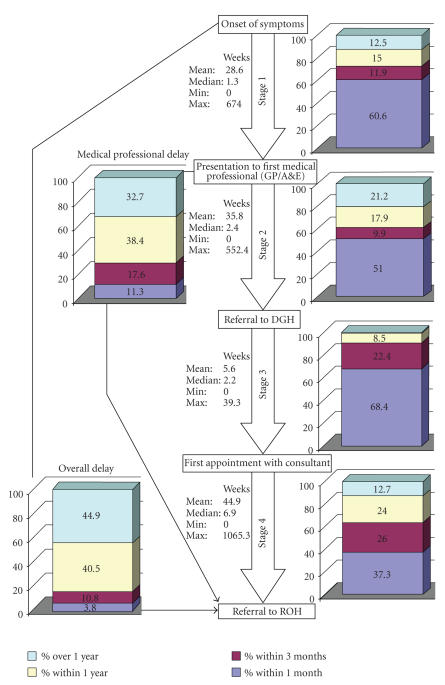
Flow chart of delay.

The previous literature highlights general practitioners as the most common source of delay in those patients who were defined as “delayed.” However, these studies identified only 20% [5] and 27% [4] of patients as delayed. Further analysis of these patients revealed that GPs were responsible in the majority of those cases. This study has demonstrated that, using median time as a method of comparison, GPs are actually quicker at referring to someone more specialised, with Figure 2 the median patient being referred within 2.4 weeks of presentation, compared with 6.9 weeks for DGH consultants. However, when examining the percentage of patients referred within 1 month and then 3 months, consultants eventually appear to be as efficient as GPs. Although only 37.3% are referred within 1 month compared to 50.1% for GPs, in a 3-month period 63.3% of patients under the care of a consultant will be referred, compared to 60.9% of patients under the care of a GP. Additionally, when comparing the percentage of patients who took longer than 1 year to be referred, it appears that DGH consultants are performing better than GPs, with 12.7% of patients taking longer than 1 year, compared to 21.2% of GP patients (Figure 3). This difference between GPs and DGH consultants is possibly due to the ordering of investigations by consultants and waiting for followup appointments. However, once the results of these investigations have been obtained, it seems that referral is prompt; the 76 patients who were given an MRI by their DGH consultant were actually referred slightly more quickly than those who were not (6.7 weeks versus 6.9 weeks) (Table 2).

It seems that the majority of GPs are referring promptly, but they are referring to the wrong specialists. However, a good proportion (40%) of GPs still takes over 3 months to refer to anyone, which is a problem that needs to be addressed. The degree of delay is somewhat dependant on the initial action of the GP. The 62 patients who were initially reassured by their GP took significantly longer to be referred than those who were not (Table 4). If they have been reassured that a lump is benign, it may then take patients a long time to represent to the GP, thereby increasing time to referral.

The figures for patients who have had a surgical intervention in a DGH are particularly interesting. It has been widely documented that surgical intervention and investigation that is not carried out in a specialist centre for sarcomas is associated with a worse prognosis in terms of survival and local recurrence. The interventions carried out are frequently incorrect, as the consultants are unaware of the diagnosis; the so-called “whoops procedure.” 48 patients had surgery at a DGH, of which 4 had a biopsy before diagnosis. 44 had no biopsy before excision, and 32 had no cross-sectional imaging. What was particularly worrying was that these patients took a mean and median 3 times (P<.001) longer to be referred to the ROH than those who were not operated on (Table 2). Therefore, not only are these patients receiving inappropriate and unnecessary surgery, but they are taking longer to be seen and treated by the specialists of choice. In most cases, the possibility of malignancy had never been considered, possibly increasing the waiting time for the operation.

We calculated BMI, recorded postal code to use the Townsend social deprivation score, and recorded the age at which patient left full-time education. The hypothesis was that patients with a higher BMI would take longer to present, as would people living in more deprived areas or with earlier age of having left full-time education. None of these factors were found to correlate with delay. The only factor that was associated with delay was that of tumour size at first presentation; as tumour size increased, time to referral to the Royal Orthopaedic Hospital decreased.

In terms of compliance with the recommended 2-week referral guidelines for suspected cancer, only 3.8% of patients were seen at the ROH within those 2 weeks. All of these patients were referred directly to the ROH from the GP; none took the typical route via a DGH. This accounts for 6 patients of the 9 patients who were referred directly to the ROH.

## 7. CONCLUSION

There is considerable medical professional delay in the management of soft tissue sarcoma. The fact that 88% of patients had at least one of the guideline features for referral to a specialist centre at first presentation suggests that knowledge of the guidelines is poor. Increased education, not only of hospital staff and general practitioners but especially at medical schools, is essential to ensure that patients are rapidly and correctly referred. The ideal pathway from GP to specialist centre needs to be emphasised, so that patients do not get referred to a DGH, where inappropriate and ineffective treatment is often used.

## Figures and Tables

**Table 1:** Histological diagnosis.

Diagnosis	Frequency	Percentage
Liposarcoma	39	24.1
Myxofibrsarcoma	21	13.0
Leiomyosarcoma	12	7.4
Spindle cell sarcoma	11	6.8
Synovial sarcoma	11	6.8
Pleomorphic sarcoma	7	4.3
MFH	6	3.7
MPNST	5	3.1
Other	50	30.9
Total	162	100.0

**Table 2:** Tumour grade and depth.

Grade	Frequency	Percentage
High	54	43.2
Intermediate	49	39.2
Low	22	17.6
Deep	61	55.5
Subcutaneous	49	44.5

**Table 3:** Summary of delays.

	Mean	Median	Min.	Max.	% in 1 month	% in 3 months	% in 1 year	% > 1 year
	Weeks							
Stage 1	28.6	1.3	0.0	674.0	60.6	72.5	87.5	100.0
Stage 2	35.8	2.4	0.0	552.4	51.0	60.9	78.8	100.0
Stage 3	5.6	2.2	0.0	208.7	68.4	90.8	99.3	100.0
Stage 4	44.9	6.9	0.0	1065.3	37.3	63.3	87.3	100.0
MP delay	83.1	25.0	0.0	1083.3	11.3	28.9	67.3	100.0
Overall time	112.3	40.4	0.4	1089.3	3.8	14.6	55.1	100.0

**Table 4:** Medical professional delay breakdown.

	Action	n=	Mean	Median	Min.	Max.	% in 1 month	% in 3 months	% in 1 year	% > year
		Weeks								
Time to first referral if MP1	Overall	162	35.8	2.4	0	552.4	51.0	60.9	78.2	100.0
	Reassures Patient	62	70.4	38.3	1.9	552.4	6.5	24.2	54.8	100
	Sends patient to A&E for X-ray	11	53.7	6.7	0	405.1	45.5	63.6	81.8	100

Time until patient referred to the ROH if 2nd MP	Overall	162	44.9	6.9	0.0	1065.3	37.3	63.3	87.3	100.0
	Reassures	3	308	185.3	79	659.4	0	0	0	100
	Performs MRI	76	28.2	6.7	0	991.3	39.5	63.2	93.4	100
	Performs biopsy	20	19.1	7.43	0.9	148.9	35	65	90	100
	Peforms biopsy and MRI	4	7.1	5.9	1.3	15.3	50	75	100	100
	Performs surgery with prior biopsy	4	69.2	22.1	2.1	230.6	25	50	75	100
	Performs surgery without prior biopsy	44	120	18.3	2.6	1065.3	9.1	29.5	75	100
	Performs surgery without prior biopsy or MRI	32	124	17.9	2.6	1065.3	9.4	37.5	71.9	100
